# Geroprotector drugs and exercise: friends or foes on healthy longevity?

**DOI:** 10.1186/s12915-023-01779-9

**Published:** 2023-12-08

**Authors:** Christian J. Elliehausen, Rozalyn M. Anderson, Gary M. Diffee, Timothy W. Rhoads, Dudley W. Lamming, Troy A. Hornberger, Adam R. Konopka

**Affiliations:** 1 Division of Geriatrics and Gerontology, Department of Medicine, University of Wisconsin-Madison, Madison, WI USA; 2 Geriatric Research, Education, and Clinical Center, William S. Middleton Memorial Veterans Hospital, Madison, WI USA; 3 Department of Kinesiology, University of Wisconsin-Madison, Madison, WI USA; 4 Department of Nutritional Sciences, University of Wisconsin-Madison, Madison, WI USA; 5 Division of Endocrinology, Department of Medicine, University of Wisconsin-Madison, Madison, WI USA; 6 William S. Middleton Memorial Veterans Hospital, Madison, WI USA; 7 Department of Comparative Biosciences, University of Wisconsin-Madison, Madison, WI USA

**Keywords:** Physical activity, Healthspan, Aging, Geroscience, Skeletal muscle, Metformin, Rapamycin, Acarbose, SGLT2 inhibitors

## Abstract

Physical activity and several pharmacological approaches individually combat age-associated conditions and extend healthy longevity in model systems. It is tantalizing to extrapolate that combining geroprotector drugs with exercise could extend healthy longevity beyond any individual treatment. However, the current dogma suggests that taking leading geroprotector drugs on the same day as exercise may limit several health benefits. Here, we review leading candidate geroprotector drugs and their interactions with exercise and highlight salient gaps in knowledge that need to be addressed to identify if geroprotector drugs can have a harmonious relationship with exercise.

## Introduction

The world is growing older. Globally, the number of people aged 65 and older is growing and is estimated to be 1.5 billion people by 2050 [1]. In the USA, the number of people reaching the age of 65 is rising and is projected to outnumber those below 18 by 2035 [2]. Aging is characterized by the progressive loss of physiological function driving increased risk for non-communicable diseases including immobility, frailty, and metabolic, cardiovascular, and neurodegenerative diseases. In the coming 2 decades, the global aging population will yield an estimated $47 trillion socioeconomic burden in healthcare expenditures [3–5]. With advancing age, the likelihood of multimorbidity increases, and therefore interventions aimed at targeting any one disease are unlikely to overcome the sequelae of other comorbidities.

Functional parameters such as cardiorespiratory fitness (CRF), daily steps, gait speed, and skeletal muscle mass, strength, and power predict the risk of morbidity and mortality in humans [6–18]. CRF, muscle mass, strength, and power alike decline with age and accelerate with each decade of adulthood with ramifications on overall metabolic health and disease risk [19–23]. Intrinsic to these functional parameters is skeletal muscle health, which includes size, contractile function, composition, and metabolism. The age-related decline in skeletal muscle health contributes to poor quality of life and is an underlying risk factor for age-associated conditions like insulin resistance, cardiovascular disease (CVD), dementia, frailty, and cancer [24, 25]. Therefore, finding interventions and molecular targets to slow or prevent the loss of physical function and skeletal muscle health is an attractive approach to reduce healthcare expenditures, delay disease onset, and improve quality of life in aging individuals.

Exercise has wide-reaching systemic effects impacting nearly every tissue and intervenes on multiple biological pathways that become impaired with age, including senescence, proteostasis, mitochondrial function/quality, nutrient signaling, DNA damage, and inflammation [26]. Through repeated exercise, these cellular and molecular changes facilitate increasing CRF, muscle mass, strength, and power while also decreasing established risk factors for cardiometabolic diseases and thereby lowering the risk of T2DM, dementia, Alzheimer’s, CVD, atherosclerosis, frailty and improving cancer survival/remission [27–29]. Despite extensive research and commercial investment, a pharmacological agent that captures the numerous pleotropic health benefits of exercise has yet to be identified; thus, efforts to increase adherence to regular exercise continues [30, 31].

This review aligns with the CDC’s consideration that exercise is a planned, structured, repetitive, and purposive physical activity. Most recent estimates identify ~ 50% of the US adult population meets aerobic physical activity guidelines (150 min of moderate to vigorous activity per week), and ~ 30% meet muscle strengthening guidelines (2x/week), while even less meet both [32]. In the USA, it is estimated only 8.7% of older adults (> 75 years of age) engage in muscle-strengthening activities [33]. Increased adherence to exercise over a lifetime has remarkable health benefits. At the musculoskeletal level, lifelong exercise delays age-related declines in functional metrics while extending a more youthful molecular phenotype later in life [34–36]. However, with increasing age, sedentary behavior and cardiometabolic risk factors (hyperglycemia, hyperlipidemia, etc.) may contribute to delayed or diminished whole body and skeletal muscle adaptive potential to exercise, which is often referred to as anabolic resistance [37–44]. Many of the proposed cellular and biological hallmarks of aging are implicated in blunting the responsiveness of skeleteal muscle to a bout of exercise [45]. However, consistent exercise can still elicit robust adaptations in older adults. One year of endurance training can improve CRF by ~ 5 ml kg^−1^ min^−1^ in previously untrained 65-year-old or older individuals [46]. Importantly, in healthy individuals, a 3.5-ml kg^−1^ min^−1^ greater CRF was associated with a 11% reduction in all-cause mortality [6]. In addition, even in adults 85 years of age and older, resistance exercise is capable of increasing muscle mass, strength, and power [47, 48]. Overall, it is never too late to engage in exercise with the intent of improving systemic and/or musculoskeletal metabolism and function to decrease mortality risk.

Intervening on conserved underlying mechanisms of aging before the development of disease could postpone the onset, slow the progression, or perhaps ameliorate multi-morbidity and extend healthy longevity. Numerous dietary, lifestyle, pharmacological, and genetic approaches have identified that lifespan is modifiable in model systems. To rigorously test proposed geroprotective treatments, the National Institute on Aging (NIA) Interventions Testing Program (ITP) was established. Based at three sites across the USA, the goal of the ITP is to evaluate whether proposed agents extend lifespan and reduce late-life diseases. The ITP uses the outbred UM-HET3 mouse, which is designed to better model the genetic diversity of humans and limit the risk of identifying interventions that apply only to strain-specific causes of death. Among the ITP and other independent groups, the mTOR inhibitor rapamycin is the most ubiquitous intervention thus far to extend lifespan in diverse species [49]. The glucose-lowering medications metformin, sodium-glucose transporter 2 inhibitors (SGLT2i), acarbose, senolytics, and estrogenic agonists (17 $-\alpha$ estradiol) have also been demonstrated by the ITP or others to extend lifespan [50–54]. Positive results from preclinical models have spurred large-scale public interest in gerotherapeutics, prompting some self-motivated individuals to take one or more putative geroprotective drugs and supplements off-label with the idea of further extending healthy longevity. Several tele-health companies have begun supplying these proposed geroprotectors to thousands of people across the globe. Importantly, it remains unclear whether the benefits of these pharmacologic approaches observed in pre-clinical models or in-patient populations extend to individuals free from overt disease who may also engage in other bona fide health-extending interventions such as exercise. Therefore, similar to the importance of determining drug-drug interactions, it is necessary to understand if the interaction between exercise and leading geroprotective drugs can have positive or detrimental impacts on the fundamental mechanisms of aging and healthy longevity.

**Fig. 1:**
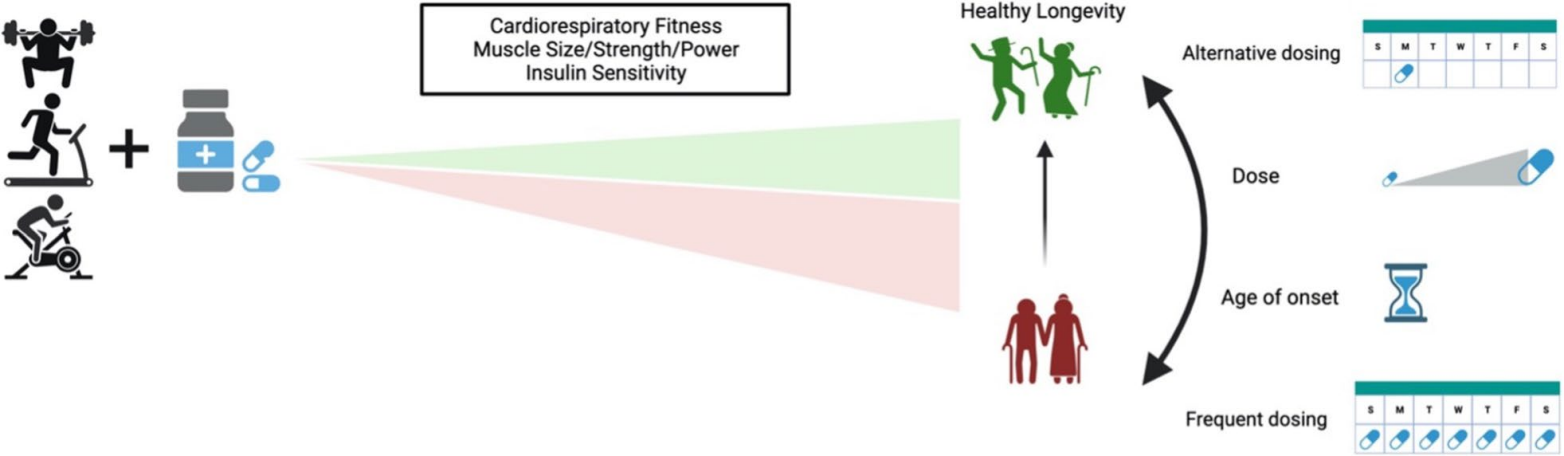
Capitalizing on the combination of regular exercise and geroprotectors. Current dogma suggests combining geroprotectors with concurrent exercise blunts hallmarks of exercise that are associated with healthy longevity. Frequent (daily) dosing of leading geroprotectors blunts clinically relevant improvements to cardiorespiratory fitness, muscle size/strength/power, and insulin sensitivity. Along the aging continuum, identifying an appropriate age to begin intervening with combined approaches represents an opportunity to suppress the age-related decline in systemic health. Finally, manipulating dose or frequency of dosing may provide the opportunity to capitalize on the benefits of both regular exercise and geroprotectors to enhance healthy longevity to new heights. Created with BioRender.com

Potentially, the combination of exercise and proposed gerotherapeutics could be used to further extend healthspan beyond either treatment alone. Here, we will briefly introduce the primary treatment indications for the mTOR inhibitor rapamycin and glucose lowering medications [55], discuss their potential impact on skeletal muscle and metabolic health, and describe current efforts investigating potential interactions between these leading geroprotectors and exercise, focusing on the likely impact on health and longevity (Fig. 1). We will also highlight potential mechanisms for consideration, discuss critical gaps in knowledge, and identify needs for future research to firmly establish whether geroprotective drugs could have a harmonious relationship with exercise.

## Rapamycin

The mTOR complex is a key regulator of cellular processes and metabolism including growth, autophagy, and nutrient signaling [56, 57]. Dysregulation of mTOR signaling disrupts cellular homeostasis and is associated with organismal aging [56]. The mTOR inhibitors rapamycin and the rapamycin analog everolimus are FDA-approved drugs with primary treatment indications for kidney transplant and some cancer patients. In pre-clinical studies in mice, rapamycin extends lifespan in both sexes and can extend lifespan even when started late and when dosed either transiently or intermittently [58–60]. Although life-phase specific and sex-adjusted dosing is an active area of investigation, current research shows that lifespan extension appears to be greatest when started earlier in life and in female mice [61]. In addition to lifespan extension, mTOR inhibitors have ameliorated many age-related conditions associated with the heart, liver, brain, skeletal muscle, and the immune system [62–67].

Despite the positive benefits on lifespan, prolonged treatment of rapamycin at doses aligned with the FDA label for immunosuppression is associated with increased risk of numerous adverse metabolic side effects, including hyperglycemia, new onset diabetes, and dyslipidemia [68, 69]. Even in a small-scale study of healthy older adults, 8 weeks of daily rapamycin (1 mg/day), which is lower than the dose used in transplant studies, tended to increase HbA1c, triglycerides, and VLDL [70]. These effects can also be seen in animal models, with IP (2 mg/kg/day) or dietary rapamycin (14 ppm) inducing hyperglycemia and insulin resistance in mice, rats, and guinea pigs [71–74]. Dietary rapamycin (14 ppm)-induced hyperglycemia was also associated with worsened osteoarthritis severity in guinea pigs [74]. In addition to metabolic side effects, rapamycin increased cataract severity in a dose-dependent fashion in both male and female UMHET3 mice [75]. Rapamycin also caused testicular atrophy in UMHET3 mice at the lowest dietary dose (4.7 ppm) and in C57BL/6 J mice when provided intermittently (once every 5 days administration (2 mg/kg)) [60, 61, 75]. So, despite the positive influence on lifespan, the incidence of rapamycin-associated side effects may oppose some aspects of healthy longevity. Therefore, it is clearly important to establish the optimal dose and dosing frequency of rapamycin and rapalogs that could be used to extend human healthspan with or without exercise while minimizing risk of adverse side effects.

Rapamycin acutely and potently inhibits mTORC1 and when given frequently for prolonged periods of time has off target inhibition on mTORC2 signaling [76, 77]. In contrast to the lifespan benefits of mTORC1 inhibition, whole body or tissue-specific inhibition of mTORC2 is largely detrimental as evident by metabolic dysfunction, frailty, and shortened lifespan in mice [76, 78–84]. Conversely, increased mTORC2 activity increases the lifespan of flies, and mTORC2 activity is elevated in long-lived Snell dwarf mice, *Ghr*^−*/*−^ mice, as well male mice treated with dietary acarbose- and 17-α estradiol [81, 85, 86]. Collectively, these data support a model by which rapamycin and rapalog mediated inhibition of mTORC1 is geroprotective, while the “off-target” inhibition of mTORC2 is responsible for several negative metabolic effects of rapamycin**.**

Due to the different kinetics of mTORC1 and mTORC2 inhibition by rapamycin, there may be a therapeutic window to maximize the health benefits associated with mTORC1 inhibition and minimize the undesirable adverse events mediated by mTORC2 inhibition by using intermittent dosing or alternative rapalogs. Intermittent rapamycin (once every 5 days) and rapalogs everolimus and DL001 enable more specific inhibition of mTORC1 with less influence on mTORC2 and decreased metabolic and immunological disruptions [60, 87]. Importantly, intermittent rapamycin treatment was able to extend lifespan in female C57BL6 mice without many of the metabolic side effects [59].

In aged skeletal muscle, mTORC1 signaling has been shown to be elevated in both preclinical models and humans [65, 88–93]. Constitutive activation of mTORC1 through genetic knockout of the upstream inhibitor TSC1 is associated with muscle atrophy and insulin resistance [94–97]. Inhibition of mTORC1 signaling by rapamycin and the rapamycin analog everolimus partially or completely preserves muscle size with increasing age and also restores or delays age-related impairments in markers of autophagy, neuromuscular junction dysfunction, muscle contractile function, grip strength, and running performance [65, 93, 98–100]. In young male mice, 2 weeks of rapamycin (2 mg/kg/day) did not impair exhaustive running performance suggesting rapamycin does not create a barrier to engage in or maintain physical activity [101]. Furthermore, knockout of the mTORC1 substrate S6K1 protects against diet-induced obesity, improves insulin sensitivity, and extends lifespan [102, 103]. In young men, a single dose of rapamycin (6 mg) inhibits mTORC1 signaling and improves skeletal muscle insulin sensitivity during hyperaminoacidemia [104]. Collectively, these studies suggest that mTORC1 inhibition by rapamycin could be viable strategy to delay the onset or slow the progression of the age-related loss of skeletal muscle health in sedentary subjects.

## Exercise + rapamycin

Both aerobic and resistance exercise can acutely increase downstream targets of mTORC1 and mTORC2 signaling, although the timing and magnitude of these effects may differ as a function of exercise mode and intensity level [105–109]. At the tissue level, changes in p70SK phosphorylation are largely mediated by mTORC1. The acute resistance exercise-induced increase in p70SK phosphorylation correlates with the extent of hypertrophy that occurs after chronic resistance exercise training, suggesting mTORC1 signaling may be related to muscle growth [110]. Studies in rodents have demonstrated that inhibition of mTORC1 by rapamycin blunts the hypertrophic response to models of chronic overload with doses as low as 0.6 mg/kg bodyweight per day in mice [111–113]. Furthermore, mTORC1 inhibition by rapamycin diminished the acute increase in mixed-muscle protein synthesis rates in both rodents and humans [114–116]. In an electrical stimulation model of resistance exercise in young, male Sprague–Dawley rats, rapamycin dampened the increase in mixed muscle protein synthesis rates after acute exercise and was associated with reduced muscle hypertrophy [116]. In addition to mTORC1, recent work has begun to elucidate the role of mTORC2 in response to muscle contractions and the regulation of muscle protein synthesis. Muscle protein synthesis can remain elevated 48 h following exercise, and the sustained elevation of muscle protein synthesis is suggested to be partially mediated by a rapamycin-insensitive and mTORC1-independent mechanism [117–119]. In mice, a combination of rapamycin and mTORC2 knockout diminished muscle protein synthesis 3 h following muscle contractions more than either condition alone [120]. Given the relationship between muscle protein synthesis and hypertrophy, it is plausible to suspect that mTORC2 contributes to hypertrophy, though this has never been formally tested. Collectively, these data suggest that along with mTORC1, mTORC2 may be necessary for the regulation of muscle growth following exercise. These data are important because they highlight how daily or continuous rapamycin administration could oppose the hypertrophic effects of exercise by inhibiting both mTORC1 and mTORC2 mediated anabolic signaling. There is an active clinical trial in middle aged to older male adults using a unilateral resistance exercise training paradigm to test if daily rapamycin (1 mg/day) impacts muscle size, strength, and proteostatic mechanisms in the resistance trained leg and/or the contralateral sedentary control leg (Clinicaltrials.gov, NCT05414292). The outcomes of this trial will be informative in guiding future exercise and rapamycin interventions.

mTORC1 and mTORC2 respond to both mechanical and nutritional cues including insulin to regulate glucose uptake [121]. The nutrient overload model of insulin resistance suggesting elevated mTORC1 signaling leads to insulin resistance via feedback inhibition of insulin/PI3K/AKT signaling from S6K [102]. Interestingly, in an electrical stimulation model of resistance exercise, the inhibition of mTORC1 with rapamycin (1.5 mg/kg) in male Sprague–Dawley rats prior to muscle contraction increases insulin stimulated skeletal muscle glucose uptake 6 h after exercise [122]. Therefore, acute inhibition of mTORC1 with resistance exercise may be a strategy to increase muscle glucose uptake more than exercise alone; however, this strategy would presumptively mitigate the increase in anabolic signaling and muscle protein synthesis. Long-term studies and different models of exercise are needed to determine if there are tradeoffs of increasing glucose uptake at the expense of restricting muscle protein synthesis. Additionally, exercise also increases known mTORC2 signaling outputs in mice and humans [108, 109] and genetic disruption of mTORC2 impaired aerobic exercise mediated glucose uptake and clearance by approximately 40% [108]. While never formally evaluated, it is possible that frequent and prolong rapamycin dosing that also inhibits mTORC2 could further interfere with several of the metabolic health benefits of exercise and limit healthy longevity.

In addition to protein and glucose metabolism, mTORC1 signaling is also involved in skeletal muscle mitochondrial bioenergetics, content, and mitophagy. In cell culture, genetic and pharmacological inhibition of mTORC1 decreases mitochondrial respiration [78, 123]; however, the impact of rapamycin treatment on skeletal muscle mitochondrial respiration in rodent muscle tissue is less consistent [101, 124–126]. This may be due to differences in mTORC1 activity in muscle at time of collection; traditionally, tissue is collected and measured following fasting which reduces mTORC1 signaling while feeding and insulin stimulate mTORC1 thereby increasing mitochondrial respiration [127]. In support, ex vivo pretreatment of muscle with leucine, an mTORC1 stimulator, increases mitochondrial respiration which is then attenuated by adding rapamycin [128].

The regulation of mitochondrial protein content by mTORC1 also has time and context dependent outcomes. mTORC1 was shown to facilitate the interaction between the transcription factor YY1 and PGC1 $\alpha$ which regulates numerous nuclear genes encoding mitochondrial proteins [124, 129]. In mice, 11 days of daily rapamycin (2.5 mg/kg) administration interrupted YY1- PGC1 $\alpha$ interaction thus reducing transcript expression of mitochondrial encoding genes in skeletal muscle [124]. In myotubes, the regulation of mitochondrial encoding genes appears time dependent, where 14 h of rapamycin acutely decreased, but 4 days of rapamycin increased expression [123, 124]. Relevant to exercise, rapamycin treatment did not suppress mRNA expression of mitochondrial genes in contracted myotubes [123]. In young female mice, a single dose of rapamycin (1.5 mg/kg) 1 h prior to moderate intensity treadmill running (1 h at 18 m/min) did not suppress markers of mitochondrial biogenesis nor crude mitochondrial protein synthesis rates; however, rapamycin did attenuate the early increase in myofibrillar protein synthesis rates [130]. These data are consistent with previous work suggesting that mitochondrial proteins may evade translational inhibition by rapamycin [131, 132]. Proteomic approaches have recently been developed to measure the protein synthesis rates of individual skeletal muscle proteins rather than crude subcellular fractions that contains hundreds of proteins. Kinetic proteomic techniques revealed that the impact of rapamycin on mitochondrial protein turnover rates are specific to individual proteins [131, 133], with the synthesis rates of many electron transport proteins decreased by rapamycin. These data indicate that rapamycin may not hinder the acute mitochondrial and metabolic responses to endurance exercise, but the long-term impact of rapamycin on oxidative and metabolic adaptations remain unknown and may differ from the acute response.

In addition to protein synthesis, inhibition of mTORC1 regulates proteostatic maintenance through autophagy, mitochondrial autophagy (mitophagy), and the ubiquitin proteasome system [94, 96, 98, 134]. With increasing age and metabolic disease, proteostatic maintenance is impaired. However, it remains unknown how the combination of exercise and rapamycin will impact proteostatic mechanisms. Interventions such as rapamycin, exercise, and caloric restriction improve proteostatic maintenance, maintain healthy mitochondrial pool, and improve skeletal muscle and organismal health [98, 134, 135]. In pathological models of mitochondrial disorders, rapamycin restores mitochondrial myopathies and delays mortality in part through regulation of autophagy/mitophagy [126, 136–139]. Interestingly, a connection between mitochondrial dynamics, mTORC1/2 signaling, and fiber type differentiation has been identified [140]. Therefore, the combination of exercise and rapamycin may be a viable strategy to augment dampened skeletal muscle plasticity in aged models [141, 142].

Due to the profound impact of rapamycin on lifespan extension in model systems, there is significant interest among the general public and the scientific community in translating these insights to human application. A recent survey study of 333 self-reported healthy and physically active adults prophylactically taking rapamycin with the goal of healthy longevity/anti-aging indicated that a fraction of rapamycin users (25–38%) reported overall improvements to quality of life related to physical health, emotional wellbeing, brain function, and aches and pains [143]. Overall, the majority of participants reported that the most common dose was 6 mg once weekly. Other reported dosing regimens were higher doses taken biweekly or lower doses taken daily. These results should be taken cautiously given the nature of self-reported bias that the authors acknowledge; however, these survey results provide additional impetus to further explore the interaction of rapamycin and rapalogs with exercise. Specifically, more work is needed to identify how the different mTORC1 and mTORC2 signaling kinetics induced by rapamycin and rapalogs might be leveraged to define a therapeutic window that capitalizes on the benefits rapamycin while minimizing antagonistic effects on exercise. Additionally, identifying populations where the greatest benefit of adding rapamycin to exercise is also of great importance. Older, insulin-resistant, and/or individuals with Alzheimer’s and related dementias often present with elevated mTORC1 signaling [65, 88–93, 144, 145], and therefore restoration to normal healthy levels could potentially improve responsiveness to exercise stimuli. However, it is incompletely understood if the benefits of rapamycin treatment on health and longevity are limited to models of increased mTORC1 signaling. More work will be needed to identify if baseline mTOR activity may contribute to the extent of healthy longevity by rapamycin. Interestingly, chronic exercise training reduces basal mTOR signaling and even lowers the magnitude of mTOR signaling following contractions on repeated training days despite increases to protein synthesis and muscle mass [146, 147]. In summary, it seems that chronic and continuous rapamycin taken in combination with exercise may not be conducive to promoting healthy longevity with regard to muscle mass and glucose tolerance and could potentially exert negative effects on other benefits of regular exercise. While speculative, it is possible that alternative dosing schedules of rapamycin with exercise presents an opportunity to capture the benefits of both interventions while minimizing negative interactions.

## Glucose lowering medications

### Metformin

Metformin is the frontline medication prescribed to patients with type 2 diabetes. Approximately 90 million prescriptions are filled each year in the USA alone [148]. Metformin has a relatively safe profile with minimal side effects; however, patients report vitamin B_12_ deficiency and gastrointestinal discomfort that typically resolves with lowering the dose [149]. Metformin suppresses hepatic glucose output; however, the benefits of metformin on the biology of aging may extend beyond glucose regulation [150, 151]. In humans and preclinical models, metformin impacts numerous cellular and molecular pathways that become dysregulated in aging such as AMPK, mTOR, inflammation, autophagy, and cellular senescence; however, these effects may be tissue and context-dependent [152, 153]. Beyond the effects on cellular processes, metformin can increase the lifespan and delay aging in model organisms, specifically nematodes and rodents with strain, sex, and dose-dependent effects [154–156]. For instance, when started at 12 months of age in male C57BL/6 and B6C3F1 mice, low dose metformin (1000 ppm in the drinking water) increased lifespan while a higher dose (10,000 ppm in the drinking water) decreased lifespan [54]. In the ITP dietary metformin (1000 ppm) did not alter lifespan in UM-HET3 mice [51]. The addition of metformin (1000 ppm) with rapamycin (14 ppm) in the diet further extended lifespan compared to historical cohorts of rapamycin alone suggesting a potential for additive benefits with cotreatments [51].

The US Diabetes Prevention Program (USDPP) demonstrated that both lifestyle modification, including 150 min of moderate intensity physical activity per week, and metformin, independently prevented the progression from prediabetes to T2DM by 58% and 31%, respectively [157]. Although the USDPP was not statistically powered for exploratory analyses, data from the cohort of older adults (> 60 years) indicates lifestyle modification decreased risk of developing T2DM by 69% while metformin did not [157, 158]. An original, retrospective analysis indicated that metformin monotherapy in patients with type II diabetes mellitus (T2DM) was associated with increased survival compared to age matched, non-diabetic controls [159]. However, a recent re-evaluation of this survival advantage in a different cohort of individuals found that metformin does not improve survival in patients with T2DM compared to either age-matched healthy controls or T2DM patients not taking metformin [160]. Therefore, caution should be used when considering the use of metformin as a geroprotective strategy because many of the proposed benefits on human aging come from preclinical models, patient populations, or those with hyperglycemia, and there is a paucity of data from people with normoglycemia and/or do not have an overt chronic disease [161]. While there was excitement for a proposal to perform the first large-scale, multisite, clinical trial to target aging with metformin (TAME), the equivocal nature of metformin on lifespan and indices of healthspan may have contributed to the stalled or delayed nature of this proposal [162]. The ongoing ANTHEM clinical trial (NCT04264897) in individuals free of disease is seeking to determine who may or may not benefit from metformin based on their antecedent metabolic health and future risk of age-related chronic conditions [163]. Results from ANTHEM and other small-scale studies could be used to inform, refine, and potentially strengthen the TAME proposal.

### *Metformin* + *exercise*

The proposition of combining metformin and exercise to further extend healthy longevity is attractive because both prescriptions improve metabolic health through multiple overlapping yet distinct pathways in various tissues [153, 164–166]. The American Diabetes Association recommends metformin and regular exercise for management of glucose in individuals with T2DM or prediabetes. However, current evidence indicates that metformin diminishes several health benefits of exercise conducive to healthy longevity in individuals without overt T2DM. In young, middle-aged, and/or older adults without T2DM, metformin inhibits the improvement in cardiovascular risk factors, insulin sensitivity, CRF, and skeletal muscle size, strength, and power [167–175]. Furthermore, metformin increases heart rate and ratings of perceived exertion during exercise which may add an additional barrier for adults to adhere to exercise guidelines [171, 176]. However, there is significant heterogeneity in the response to metformin during exercise. Our retrospective analyses identified subjects with the greatest insulin sensitivity and highest mitochondrial complex-1 respiration at baseline had no improvement or a decrease in insulin sensitivity when taking metformin with exercise [163]. Conversely, those individuals who were relatively less metabolically healthy did not experience the inhibitory or detrimental effects of metformin [163]. These findings are consistent with observations by Knowler et al., indicating those with greater baseline BMI or HbA1c had a greater decrease in the relative risk reduction for T2DM following metformin treatment [157]. It is important to note that most studies to date have investigated metformin administered daily at clinically relevant doses (1500–2000 mg/day). Therefore, it remains unknown if a different dose or dosing schedule could work with exercise to promote healthy longevity.

The mechanisms by which metformin antagonizes the cardiometabolic benefits of exercise remain largely unknown. Understanding how metformin inhibits several exercise adaptations may unlock clues on how to co-prescribe metformin and exercise to have additive or at least not detrimental effects. In response to aerobic exercise training, metformin completely abrogated the improvements in skeletal muscle mitochondrial complex I linked respiration [170]. Therefore, it is reasonable to suspect that restricting the ability of mitochondria to meet the increased energetic demands of exercise would create significant cellular energetic stress and stimulation of AMPK. However, studies investigating the interaction of metformin and exercise have demonstrated equivocal results ranging from metformin inhibiting, having no effect, or increasing skeletal muscle AMPK signaling after acute or chronic exercise [168, 170, 176]. The exercise mode, duration, intensity, and biopsy timing may all play a role in the discordant results. In muscle biopsy samples from the MASTERS trial, metformin increased the phosphorylation of acetyl-CoA carboxylase a downstream target of AMPK after 14 weeks of resistance exercise training, and this was accompanied by a trend to attenuate the increase in mTORC1 signaling [175]. Similarly, in human primary myotubes, metformin (10 mM) increased AMPK signaling and inhibited the increase in mTORC1 signaling after electrical stimulation. A follow-up analysis of the MASTERS trial using transcriptomics revealed metformin blunted the number of differentially expressed genes in skeletal muscle by ~ 30% (PLA—2048 vs MET—1435) [177]. Subsequent analyses identified that metformin may positively intervene on several aging associated pathways; however, these were not related to increased skeletal muscle size or maximal power production that are linked to clinically important outcomes. We also have unpublished data in adult male mice suggesting metformin attenuates the improvement in glucose tolerance, skeletal muscle mitochondrial respiration, and the number of differentially expressed genes following treadmill exercise training. In addition, while metformin alone was not tested, the addition of metformin to rapamycin treatment further suppressed cumulative mitochondrial and whole muscle protein synthesis more than rapamycin alone suggesting a role for metformin to alter proteome remodeling and turnover [133]. The energetic stress that accompanies metformin treatment may restrict changes to gene expression and proteome turnover to attenuate exercise induced cellular remodeling and adaptation.

In summary, it is unknown whether metformin can be combined with exercise to maintain or slow the age-related loss of health. Moreover, the heterogeneous responses to metformin provide challenges and opportunities to unravel the mechanisms underlying the antagonistic or positive effects of metformin on several health benefits of exercise. For example, vitamin B_12_ is a critical co-enzyme in both the mitochondria and cytosol to maintain substrate metabolism, and it remains unknown if those who experience vitamin B_12_ deficiency are more susceptible to the inhibitory role of metformin on whole body or skeletal muscle adaptations to exercise. Traditionally, metformin is taken once or twice daily and has a plasma half-life of ~ 6 h but can remain in cells and tissues for up to 24 h. Hypothetically, this presents an opportunity to test whether alternating days of exercise and metformin could be implemented to avoid unwanted side effects and capture the health benefits of both prescriptions.

### SGLT2 inhibitors

SGLT2 inhibitors (SGLT2i) such as empagliflozin, canagliflozin, and dapagliflozin have been identified as a pharmacological alternative to metformin. SGLT2i lower glucose by attenuating glucose uptake in the kidneys causing glucosuria [178]. Like metformin, there is a plethora of data in prediabetic and diabetic populations to indicate that SGLT2i monotherapy helps regulate glucose and manage HbA1c which are accompanied by decreased CVD events, improved vascular function, weight loss, substrate utilization, and improved cardiac function [179–183]. In patients with heart failure and preserved ejection fraction, empagliflozin reduced cardiovascular death or hospitalization for heart failure independent of diabetes [184]. In the ITP, dietary canagliflozin (180 ppm) started at 7 months of age extended the lifespan of UM-HET3 male mice by 14% with no effect on females despite lower fasting glucose and improved glucose tolerance in both sexes [52]. Concerningly, canagliflozin use in patients with T2DM, UM-HET3 mice, and rats report adverse effects on bone health as evident by decreased bone mineral density and increased risk of fracture [185–189].

### *SGLT2i* + *exercise*

Currently, there are few studies that have investigated the combination of SGLT2i and exercise. Young Sprague–Dawley rats were fed a high fat diet for 12 weeks and treated with streptozotocin to induce obesity and mimic T2DM. Following the 12-week high-fat diet plus streptozotocin lead in, the co-treatment of canagliflozin (3 mg/kg/day) and 12-week treadmill running (60 min/day, 5 days/week) significantly reduced body weight gain to a greater extent than exercise alone while equally improving glucose tolerance, submaximal exercise performance, and increased reliance on fat as a fuel source [190]. To investigate whether SGLTi can restore the adaptative response to exercise due to correction of hyperglycemia independent of overt body weight differences, streptozotocin-induced hyperglycemic male CD-1 mice were treated with the combination of canagliflozin (~ 30 mg/kg/day) and 8 weeks of voluntary wheel running [191]. The cotreatment of exercise and SGLT2i was able to restore fasting blood glucose, and glucose tolerance during an oral glucose tolerance test, and improve VO_2_ max greater than exercise alone in hyperglycemic mice [191]. These metabolic and physical adaptions were associated with an increased oxidative fiber type distribution and capillary density. Importantly, this study employed normoglycemic (no streptozotocin) exercise-trained mice, and the improvements to glucose tolerance, mitochondrial content, body composition, oxidative fiber type shift, and capillary density were equivalent to hyperglycemic mice co-prescribed SGLT2i and exercise. These data support the notion that restoration to normoglycemia with SGLT2i is a viable strategy with exercise to further capitalize on the health benefits of aerobic exercise.

In patients with T2DM, the combination of exercise and SGLT2i is more effective at improving metabolic health than SGLT2i treatment alone [192]; however, there is limited data in individuals without T2DM. In overweight or obese men and women (18–50 years old) with normal fasting glucose, adding daily dapagliflozin (5–10 mg/day) to aerobic exercise training for 12 weeks surprisingly increased baseline fasting blood glucose and blunted the improvement in whole body insulin sensitivity after exercise training [193]. However, cotreatment of dapagliflozin and exercise did not impact the exercise-induced improvements to body composition, VO_2_max, or indices of mitochondrial function. More work is needed exploring the interaction between various exercise modalities and SGLT2i to determine if SGLTi can have favorable effects on exercise adaptations. Furthermore, there is a need to understand whether the osteogenic effects of exercise can protect against the decline in bone health with SGLT2i use [185–189]. The limited data to date in humans suggests SGLT2i may interfere with glucose control and insulin sensitivity but do not negatively impact several other health benefits of exercise. However, there is an urgent need for more data, particularly in non-patient populations undergoing different exercise regimens.

### Acarbose

Acarbose inhibits intestinal $\alpha$-glucosidase to delay the digestion of polysaccharides thus attenuating uptake of glucose in the GI tract and lowering postprandial glucose excursions. Overall, the impact of dietary acarbose on lifespan extension appears to be sex-, age-, and dose-dependent in UM-HET3 mice. When started at 4 months of age, dietary acarbose extends lifespan of male and female mice by 22% and 5% respectively, but when started at 16 months, it only extends lifespan in male mice by 6% [51, 194]. Furthermore, in females, the highest acarbose dose (2500 ppm) has a greater lifespan extension than 1000 ppm [53]. The lifespan extension in males by acarbose has been largely attributed to not only reduced neoplastic disease but also a reduction in mTORC1 signaling and cap-independent translation, a shared trait among long-lived models [195]. Furthermore, acarbose (1000 ppm) increased mTORC2 signaling in the liver and improved glucose tolerance in male mice [85]. Additionally, similar to metformin, acarbose when combined with rapamycin starting at 9 months of age extends lifespan even further than rapamycin alone with the largest reported increase in median lifespan by the ITP at 28% for females and 37% for males [196]. Acarbose may have additional benefits to the cardiovascular system; however, it is unclear if these results are secondary to the glucose-lowering properties [197]. Recent evidence suggests a portion of the population may be resistant to acarbose via a mechanism that involves microbiota-based degradation of acarbose [198]. Efforts to circumvent the degradation of acarbose may be necessary to broadly translate acarbose into a geroprotector for healthy longevity.

### *Acarbose* + *exercise*

The combination of acarbose and exercise is a limited area of research, particularly in populations without T2DM. In 8-week-old C57BL/Ks (db/db) mice, daily acarbose (40 mg/kg/day) combined with 4 weeks of swimming exercise did not abrogate any exercise-induced benefits to fasting glucose [199]. In patients with T2DM (50–58 years old) with 60% already taking antidiabetic drugs, the addition of daily acarbose (100 mg 3 times per day) and 12 weeks of moderate-intensity aerobic exercise yielded improvements to fasting glucose, HbA1c, insulin sensitivity, HbA1C, and VO_2_max while exercise alone was only capable of improving insulin sensitivity [200]. These findings demonstrate the potential of combining acarbose and exercise to further improve metabolic health in subjects with T2DM. However, due to the limited number of studies so far, it remains unknown if adding acarbose to exercise could further yield healthspan or lifespan-extending effects in non-patient populations. The exploration of combining exercise and acarbose will be a fruitful area of research. Since acarbose works differently than metformin or SGLT2 inhibitors, it may be possible to exploit the benefits of both interventions in tandem.

## Conclusions and limitations

**Fig. 2:**
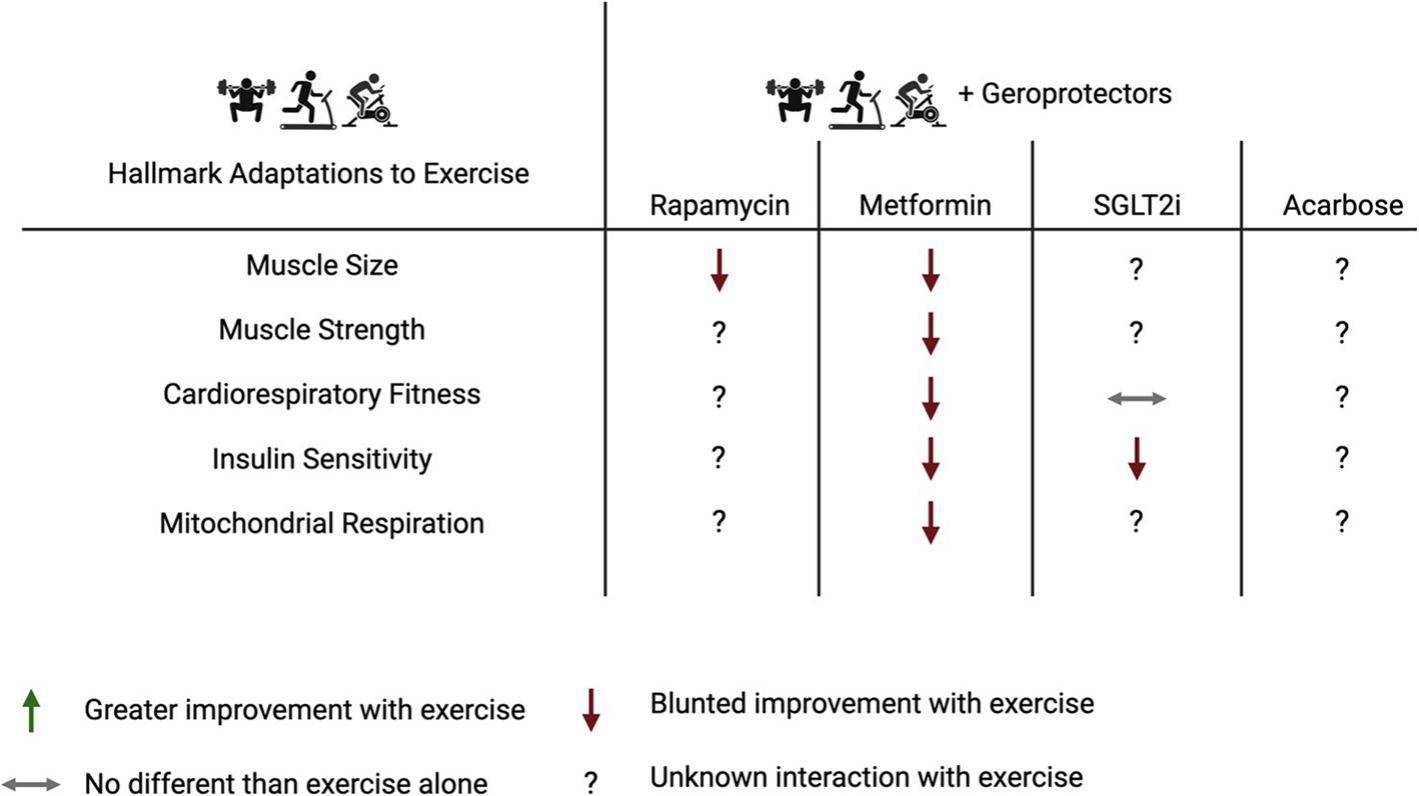
Current known and unknown interactions between leading geroprotectors and chronic exercise adaptations. Compared to regular exercise, frequent dosing of leading geroprotectors with concurrent exercise blunts many hallmark adaptions to exercise in populations without overt disease. Green up arrow, greater improvement with exercise training; red down arrow, blunted improvements with exercise; horizontal grey arrow, no different than exercise alone; question mark, indicates unknown interaction. Created with BioRender.com

Several pharmacological interventions have been effective at combating aging hallmarks, ameliorating aging diseases, and extending lifespan in preclinical models. Exercise is one of the most impactful lifestyle modifications that can decrease the risk of many cardiometabolic diseases and some cancers in humans. Exercise modulates several fundamental mechanisms of aging and may have rejuvenating aspects in aging tissues [201, 202]. The existing evidence suggests that most leading geroprotective drugs do not cooperate with concurrent exercise training and may limit the healthspan extending effects of exercise (Fig. 2). Opportunities for future research are ripe given few have assessed alternative dosing schemes in the attempt to harness the benefits of exercise and geroprotectors to modulate the biology of aging harmoniously.

This review is focused on select geroprotectors that were recently highlighted to have the highest potential benefit based on the limited available data [55]. However, there are other compounds, such as senolytics and 17 $-\alpha$ estradiol, that also have translational potential in preserving metabolic health and physical function with age [50, 85, 203]. Considering that each putative geroprotector and exercise may have sex-specific effects on health and longevity [204–206], a limitation to date is that most studies investigating the interaction of exercise and geroprotectors have used male models. Therefore, the inclusion of females and rigorous study into the biological impact of sex is strongly needed when investigating the interaction of proposed geroprotectors and exercise.

We acknowledge this review largely focused on skeletal muscle due to its critical role in modulating systemic health with increase age. However, the interaction of aging, exercise training, and proposed gerotherapeutics also occur in the heart, liver, brain, adipose, cardiovascular, immune, nervous system, and brain. Studying alternative tissues and the relationship to healthy longevity in response to geroprotector drugs and exercise is also of great interest.

This review focuses largely on clinically relevant functional metrics such as muscle strength and cardiorespiratory fitness because they are relatively easy and inexpensive to perform and are well validated and strongly predictive of morbidity and mortality [6–18]. While there is great interest and need to identify biomarkers of aging [207], there is currently a lack of consensus on validated biomarkers that outperform or strengthen existing functional or clinical outcomes. The use of biomarkers to inform on the clinical benefits or consequences of geroprotector drugs plus exercise is an exciting area for future research.

Consistent with the goal of healthy longevity, there are several outstanding questions (Table 1) that should be considered before the broad implementation and prophylactic use of potential geroprotector drugs in individuals who are free of disease and/or physically active. We aim through this review to encourage future research to evaluate the interaction of proposed geroprotectors with regular exercise across the spectrum of age groups and antecedent metabolic health. If regular exercise and proposed geroprotectors can be determined to work harmoniously, then perhaps we may find more effective strategies to extend healthy longevity.

**Table 1:** Outstanding questions

What age is considered optimal for adding a geroprotector to exercise?
Does antecedent health identify populations that should or should not begin adding geroprotectors to exercise?
Can a geroprotector be given at the onset of a new exercise regimen in an otherwise inactive person to “jumpstart” health benefits?
How long should the geroprotector be used?
What dose, time of day, administration route, and schedule (transient, monthly, biweekly, weekly, daily) are most favorable to potentiate the health benefits of exercise?

## Data Availability

Not applicable.
